# The association between psychological distress and angina pectoris: A population-based study

**DOI:** 10.1371/journal.pone.0224451

**Published:** 2019-11-08

**Authors:** Ching-Ching Tsai, Shao-Yuan Chuang, I-Chang Hsieh, Lun-Hui Ho, Pao-Hsien Chu, Chii Jeng

**Affiliations:** 1 Department of Nursing, College of Nursing, Chang Gung University of Science and Technology, Tao Yuan, Taiwan; 2 Department of Cardiology, Chang Gung Memorial Hospital, Linkou, Taiwan; 3 Institute of Population Health Sciences, National Health Research Institutes, Miaoli, Taiwan; 4 Department of Cardiology, Heart Failure Center, Chang Gung Memorial Hospital, Linkou, Taiwan; 5 College of Medicine, Chang Gung University, Tao Yuan, Taiwan; 6 Department of Nursing, Chang Gung Memorial Hospital, Linkou, Taiwan; 7 School of Nursing, College of Nursing, Taipei Medical University, Taipei, Taiwan; Maastricht University Medical Center, NETHERLANDS

## Abstract

**Background:**

Psychological distress is an undifferentiated combination of symptoms that may be related to the occurrence of angina pectoris (AP). However, few studies have investigated the relationship between psychological distress and AP, particularly in Asian populations. The purpose of this study was to examine the relationship between psychological distress and AP in Taiwanese adults.

**Methods:**

We adopted a cross-sectional design to explore the data of the 2005–2008 Nutrition and Health Survey in Taiwan. In total, 2080 subjects (aged ≥ 19 years) responded to questionnaire interviews and underwent physical examinations. Each of the five dimensions of psychological distress (sleep disturbance, anxiety, hostility, depression, and feelings of inferiority) were scored (from 0–20) according to the Five-Item Brief Symptom Rating Scale (BSRS-5). A score ≥ 6 points indicated psychological distress. AP was evaluated using a modified Rose questionnaire.

**Findings:**

In total, 102 subjects (3.6%) had AP, and 231 subjects (8.8%) had symptoms of psychological distress. After adjusting for the basic data, metabolism, and lifestyle covariates, the BSRS-5 total score was associated with AP (odds ratio [OR] = 1.2, 95% confidence interval [CI] = 1.13–1.26, *p* < 0.001). Subjects with psychological distress had a higher risk of AP (OR = 2.97, 95% CI = 1.76–4.99, *p* < 0.001).

**Conclusions:**

The presence of AP is associated with psychological distress. Health care providers should therefore be aware of the impact of psychological distress on AP. Our study findings can serve as a reference for AP assessments. Large scale longitudinal studies are needed to confirm a causal relationship between psychological distress and AP.

## Introduction

Psychological distress involves changes in emotional state, discomfort, or harm experienced because of an individual’s inability to effectively respond to, or control, life pressures or unmet demands. The prevalence rate is between 5% and 27% in the general population [1]. Psychological distress generally manifests as anxiety, depression, hostility, insomnia, or anger, which varies depending on personal traits and culture. These negative characteristics usually overlap, leading to the occurrence of symptom clusters [1,2]. It has been suggested that multidimensional tools for comprehensive assessment are necessary to prevent underestimation of the burden of psychological distress.

Psychological distress through biological and behavioral pathways affects the incidence, progression, and mortality rates of many chronic illnesses, especially cardiovascular conditions [3,4], including exacerbation of the occurrence and frequency of angina pectoris (AP) symptoms [5–7]. A meta-analysis across 31 countries indicates that the prevalence of AP ranges from 0.7% to 15% in the general population [8]. AP is a common symptom of coronary artery disease (CAD), with myocardial hypoxia arising from obstructive or non-obstructive CAD; it can also result from non-CAD conditions (such as anemia, hyperthyroidism, respiratory diseases, and valvular disease), that may independently predict the incidence rates of myocardial infarction (MI) and cardiovascular diseases after adjustment for age [9]. Furthermore, gastrointestinal disorders may result in non-cardiac chest pain that mimics AP through changes in pain sensitivity and stimuli by vagal nerve fibers shared with the heart [10,11].

Previous studies of the psychological factors related to AP explored more than five factors from a single perspective [12–14]. However, separate evaluation of various single factors may increase the length of the questionnaire to the point that clinical evaluation or screening is difficult. Moreover, the majority of community-based studies on factors impacting AP focused on traditional CAD risks, including demographic, metabolic, and lifestyle factors, and overlooked the influence of other physiological conditions that might cause myocardial ischemia, shared symptoms, or comorbidities [15–18]. Few studies have considered broadly associated factors (such as anemia, respiratory, and gastrointestinal diseases) that might elucidate a relationship between overall psychological distress and AP, particularly in Asian populations [19,20]. Therefore, the purpose of this study was to examine the correlation between psychological distress and AP by using Taiwan’s national population survey data.

## Materials and methods

### Data sources and subjects

This study adopted a cross-sectional comparative design using data from the Nutrition and Health Survey in Taiwan (NAHSIT), a nationwide population-based survey sponsored by the Ministry of Health and Welfare of Taiwan. The NAHSIT continually monitors nutrition and health parameters. Six surveys have been completed since 1980; the results of the sixth survey (conducted between 2013 and 2016) remain unreleased and we therefore analyzed the data obtained from the fifth NAHSIT survey (conducted between 2005 and 2008) [21]. This study was conducted after approval from the Institutional Review Board of Chang Gung Medical Foundation (IRB No. 104-9035B).

In the 2005–2008 NAHSIT, the 358 townships and city districts in Taiwan were divided into five geographical and three cultural strata. Two strata were subjected to three-stage probability sampling, and a total of 48 townships and city districts were selected. Three visits (from March to June, July to October, and November to February) were performed each year to control for seasonal effects. First, the facilitator communicated with recruitment area authorities to determine the locations and times of home visits and health examinations. Subsequently, a well-trained interviewer explained the study procedure to the subjects before a written consent form was completed. Questionnaires were administered using a standardized process. The questionnaire collected basic demographic information, medical data (such as medical and medication histories, brief symptom rating scale, and angina history), and lifestyle information (such as smoking, diet, and physical activity). A computer-assisted personal interview method was used to improve efficiency and reduce recording errors. Finally, the blood pressure, waist circumference, height, and weight of the subjects were collected during the health examination. Subjects provided blood samples after fasting for more than 8 h, and these were collected in different collection tubes and sent to a laboratory for centralized analysis. Overall, 4686 individuals aged ≥ 19 years were interviewed, but only 2808 subjects who consented to receive the physical examination [21,22] were included in this study.

### Definition of AP

The AP questionnaire used in the NAHSIT was a modified Rose Angina Questionnaire [23] recommended by the World Health Organization for epidemiological surveys. The original version of the questionnaire was developed in 1962 to assess triggering and mitigating factors as well as the location, frequency, nature, severity, and duration of chest pain [24]. Compared to clinical diagnoses, exercise echocardiograms (ECGs), and myocardial perfusion scans, the questionnaire had a sensitivity of 53–86% and specificity of 70–89% [25,26].

The AP duration was not assessed in the NAHSIT nine-item AP questionnaire, and the frequency and age at the first occurrence of AP were not considered in this study. We selected the following five symptoms for evaluation, and if a case satisfied all conditions, it was considered AP-positive: 1) chest pain or discomfort occurred at least once; 2) chest pain or discomfort was felt when walking fast or climbing a slope; 3) slowing down or stopping alleviated chest pain or discomfort; 4) chest pain or discomfort disappeared while resting; and 5) the areas of pain or discomfort included any area of the chest (including the left, center, right, upper, and lower chest) [17,18].

### Measurement of psychological distress

The Five-Item Brief Symptom Rating Scale (BSRS-5) was used in this study to assess psychological distress. This scale, initially developed by Lee et al., [27] was taken from the 50-item Brief Symptom Rating Scale (BSRS-50). It assesses trouble falling asleep; feeling tense or “keyed up” (i.e., anxious); feeling easily annoyed or irritated; feeling depressed, blue, or sad; and feeling inferior to others in the past week. These feelings represent the following five dimensions: sleep disturbance, anxiety, hostility, depression, and inferiority, respectively. The questionnaire is scored based on the five-point Likert scale, with 0 indicating “not at all”; 1, “mild”; 2, “moderate”; 3, “severe,” and 4, “very severe.” The score range is 0–20 points, and a total of 5–6 out of 20 points is considered the cutoff value for the early detection of psychiatric disorders. BSRS-5 was shown to exhibit high degrees of reliability and validity in Taiwan [27–29]. A BSRS-5 total score of ≥ 6 was defined as psychological distress. The value of Cronbach’s *α* in this study was 0.85.

### Measurement of covariables

In this study, we extensively screened candidate factors affecting the incidence of AP from the NAHSIT database and classified them into three major categories: basic information, metabolism, and lifestyle. Basic information included basic characteristics, medical history, and medication history. We first asked the subjects about their employment—whether they currently had a full- or part-time job. Further, we enquired about their economic status using the following question: “Do you have enough money in your family to meet your monthly living expenses and other expenses?” If the answer was “enough,” “there is extra money,” or “just enough without difficulty,” the subject’s economic status was denoted as “enough”; if the answer was “somewhat difficult” or “difficult,” then the subject’s status belonged to the “difficult” category. We also asked “How do you think your health status is compared with that of other people of the same age and gender?” If the answer was “much better than others,” “better,” or “similar,” then the perceived health status was denoted as “good”; if the answer was “a little worse” or “much worse than others,” then it was denoted as “poor.” Medical history included diseases diagnosed by a physician, as reported by the subject, and medication history included the medications taken in the last month, as reported by the subject.

Metabolic data were obtained from the results of health examinations and blood tests (including C-reactive protein, uric acid, and hemoglobin levels). Body mass index was calculated as weight (kg)/height (m)^2^. Central obesity corresponded to a waist circumference of ≥ 90 cm for males and ≥ 80 cm for females. Blood pressure was determined from the average of four measurements, with high blood pressure corresponding to a systolic blood pressure of ≥ 130 mmHg and diastolic blood pressure of ≥ 85 mmHg or the use of antihypertensive drugs. Hyperglycemia corresponded to a fasting blood glucose value of ≥ 100 mg/dl or the use of hypoglycemic drugs. The high-density lipoprotein level was considered low at a concentration of < 40 mg/dl for males and < 50 mg/dl for females. Hypertriglyceridemia corresponded to a triglyceride level of ≥ 150 mg/dl. Three or more of the five factors mentioned above indicated the presence of metabolic syndrome [30].

In the lifestyle section, physical activity involved walking, running, mountain climbing, dancing, or swimming at least once a week in the past year. Having smoked no more than 100 cigarettes over the subject’s lifetime was considered “never smoked”; having smoked more than 100 cigarettes and having quit smoking was considered “former smoker”; and current smokers were categorized as “currently smoking.” Those who self-declared that they never consumed alcohol or an alcoholic drink were considered to “never drink alcohol”; those who did not drink alcohol regularly were considered to “occasionally drink alcohol”; and those who drank alcohol regularly were considered to “often drink alcohol.” The food frequency questionnaire was used to assess whether coffee, tea, or dietary supplements had been consumed in the past month; this questionnaire’s reliability and validity were also confirmed [31]. A 24-h dietary recall was conducted to record all food types and the food intake volume for the past 24 h. After analysis, we were able to determine the approximate number of calories and amounts of various nutrients consumed by the subject in the last 24 h; the reliability and validity of the data have been confirmed for this method as well [32]. In this study, each subject’s cholesterol and saturated fatty acid (SFA) intake was divided by the number of total calories to calculate the nutrient density. This value was multiplied by 1500 kcal to obtain the adjusted cholesterol and SFA intake. The fraction of calories corresponding to SFA was calculated as the percentage of the total calories as follows: (adjusted SFA intake × 9 / 1000) / 1500 × 100 [31].

### Data analysis

Data analyses were performed using SAS 9.3 statistical software (SAS Institute, Cary, NC, USA). The released data were checked for unreasonable values, and anonymized. AP occurrence rate, basic information, metabolism, lifestyle, and psychological distress occurrence rate were described in terms of both the raw number and percentage of subjects. Distribution state of continuous variables was examined with the Kolmogorov-Smirnov test. Normally distributed variables are presented as mean and standard deviation; non-normally distributed variables are reported as median and interquartile range (IRQ). A chi-square test was used to compare the basic characteristics of the respondents who participated in both an interview and health examination versus non-respondents who participated in an interview but not an examination. Simple logistic regression analysis was employed to calculate the odds ratios (OR) and 95% confidence intervals (CI) to assess the relationship between candidate factors and AP. In the multiple logistic regression model, in addition to age and sex, we included variables with *p* < 0.15 during the simple regression analysis to avoid omitting possible confounding factors [33]. In BSRS-5, a stepwise logistic regression with continuous (total score) or discrete (scores < 6 and ≥ 6) variables was performed to construct an AP prediction model. For the two-tailed test, *p* < 0.05 was considered statistically significant.

## Results

No differences were found between the respondents and non-respondents for sex, ethnicity, geographic location, or economic status. However, respondents tended to be older and were more likely to be unemployed than non-respondents (supplemental S1 Table).

The median BSRS-5 total score was 1 point (IRQ = 0–3 points) in the 2808 NAHSIT respondents enrolled in this study; of these, 102 (3.6%) had AP and 231 (8.8%) had a total BSRS-5 score ≥6. Among the subjects with AP, 61.8% were female, and 25% exhibited psychological distress. Tables 1 and 2 illustrate the correlation between candidate factors (basic information, metabolism, lifestyle and psychological distress) and AP.

**Table 1:** Simple logistic analysis of basic information on the subjects with and without angina pectoris (AP).

Variable	Total	AP (+)	AP (–)	OR (95% CI)	*p*
	*N* = 2808	*N* = 102	*N* = 2706		
	*n* (%)	*n* (%)	*n* (%)		
Basic characteristics					
Sex					
Male	1375 (49.0)	39 (38.2)	1336 (49.4)		
Female	1433 (51.0)	63 (61.8)	1370 (50.6)	1.6 (1.1–2.4)	0.028
Age (years)					
19–44.9	840 (29.9)	28 (27.5)	812 (30.0)		
45–64.9	945 (33.7)	33 (32.3)	912 (33.7)	1.1 (0.6–1.8)	0.854
≥ 65	1023 (36.4)	41 (40.2)	982 (36.3)	1.2 (0.7–2.0)	0.444
Ethnicity					
Fukienese	1612 (57.4)	49 (48.0)	1563 (57.7)		
Hakka	572 (20.4)	21 (20.6)	551 (20.4)	1.2 (0.7–2.1)	0.462
Other	624 (22.2)	32 (30.4)	592 (21.9)	1.7 (1.1–2.7)	0.019
Geographic location					
Northern	1066 (38.0)	36 (35.3)	1030 (38.1)		
Central/Southern	729 (26.0)	27 (26.5)	702 (25.9)	1.1 (0.7–1.8)	0.712
Eastern/Other	1013 (36.0)	39 (38.2)	974 (36.0)	1.2 (0.7–1.8)	0.564
Marital status					
Unmarried	320 (11.4)	7 (6.9)	313 (11.6)		
Married with living spouse	1994 (71.0)	71 (69.6)	1923 (71.0)	1.7 (0.8–3.6)	0.211
Other	494 (17.6)	24 (23.5)	470 (17.4)	2.3 (1.0–5.4)	0.058
Education					
Primary school and below	1187 (42.3)	47 (46.1)	1140 (42.1)		
Junior/senior high school	1059 (37.7)	40 (39.2)	1019 (37.7)	1.0 (0.6–1.5)	0.823
College and above	562 (20.0)	15 (14.7)	547 (20.2)	0.7 (0.4–1.2)	0.176
Employed, yes (*n* = 2775)	1218 (43.9)	46 (45.1)	1172 (43.8)	1.1 (0.7–1.6)	0.803
Economic status, difficult (*n* = 2712)	839 (30.9)	37 (37.0)	802 (30.7)	1.4 (0.9–2.0)	0.159
Perceived health status, poor (*n* = 2713)	678 (18.5)	46 (46.0)	632 (24.2)	2.7 (1.8–4.0)	<0.001
Medical history, yes					
Stroke	86 (3.1)	2 (2.0)	84 (3.1)	0.6 (0.2–2.6)	0.514
Heart disease	239 (8.5)	32 (31.4)	207 (7.6)	5.5 (3.6–8.6)	<0.001
Hypertension	680 (24.2)	36 (35.3)	644 (23.8)	1.8 (1.2–2.7)	0.009
Respiratory tract	233 (8.3)	16 (15.7)	217 (8.0)	2.1 (1.2–3.7)	0.007
Gastrointestinal tract	633 (22.5)	30 (29.4)	603 (22.3)	1.6 (0.9–2.3)	0.093
Thyroid disease	104 (3.7)	5 (4.9)	99 (3.7)	1.4 (0.5–3.4)	0.514
Diabetes	263 (20.0)	11 (10.8)	252 (9.3)	1.2 (0.6–2.2)	0.617
Hyperlipidemia	240 (8.5)	13 (12.7)	227 (8.4)	1.6 (0.9–2.9)	0.126
Gout	242 (8.6)	15 (14.7)	227 (8.4)	1.9 (1.1–3.3)	0.028
Depression	40 (1.4)	2 (2.0)	38 (1.4)	1.4 (0.3–5.9)	0.642
**Medication used, yes**					
Heart disease	200 (7.1)	23 (22.5)	177 (6.5)	4.2 (2.6–6.8)	<0.001
Hypertension	609 (21.7)	33 (32.4)	576 (21.3)	1.8 (1.2–2.7)	0.009
Respiratory disease	51 (1.8)	1 (1.0)	50 (1.8)	0.5 (0.1–3.9)	0.527
Gastrointestinal disease	186 (6.6)	18 (17.6)	168 (6.2)	3.2 (1.9–5.5)	<0.001
Thyroid disease	15 (0.5)	2 (2.0)	13 (0.5)	4.1 (0.9–18.6)	0.064
Diabetes	239 (8.5)	10 (9.8)	229 (8.5)	1.2 (0.6–2.3)	0.634
Hyperlipidemia	118 (4.2)	7 (6.9)	111 (4.1)	1.7 (0.8–3.8)	0.178
Gout	109 (3.9)	8 (7.8)	101 (3.7)	2.2 (1.0–4.6)	0.039
Anxiolytics/hypnotics	95 (3.4)	4 (3.9)	91 (3.4)	1.2 (0.4–3.3)	0.760
Analgesics	177 (6.3)	11 (10.8)	166 (6.1)	1.9 (1.0–3.5)	0.062

OR, odds ratio; CI, confidence interval.

**Table 2:** Simple logistic analysis of metabolism, lifestyle, and psychological distress characteristics of subjects with and without angina pectoris (AP).

Variable	Total	AP (+)	AP (–)	OR (95% CI)	*p*
	*N* = 2808	*N* = 102	*N* = 2706		
	*n* (%)	*n* (%)	*n* (%)		
**Metabolic, yes**					
Body-mass index (kg/m^2^) ^a^	24.2 (21.7–26.8)	24.5 (4.1)	24.2 (21.7–26.8)	1.0 (1.0–1.1)	0.984
Waist circumference (cm) ^a^	83.4 (75.5–91.0)	82.8 (11.6)	83.5 (75.5–91)	1.0 (1.0–1.0)	0.668
Central obesity	1177 (42.8)	45 (46.4)	1132 (42.6)	1.2 (0.8–1.8)	0.463
High blood pressure	1088 (38.7)	39 (38.2)	1049 (38.8)	1.0 (0.7–1.5)	0.914
Hyperglycemia	1544 (55.0)	48 (47.1)	1496 (55.3)	0.7 (0.5–1.1)	0.103
Lower high-density lipoprotein	748 (27.8)	32 (33.0)	716 (27.6)	1.3 (0.8–2.0)	0.243
Hypertriglyceridemia	756 (28.1)	26 (26.8)	730 (28.1)	0.9 (0.6–1.5)	0.779
Metabolic syndrome	959 (34.2)	31 (30.4)	928 (34.3)	0.8 (0.6–1.3)	0.415
C-reactive protein (mg/dL) ^a^	0.1 (0.1–0.2)	0.1 (0.1–0.3)	0.1 (0.1–0.2)	1.0 (0.8–1.4)	0.780
Uric acid (mg/dL) ^a^	5.8 (4.8–7.1)	6.2 (1.8)	5.8 (4.8–7.1)	1.1 (1.0–1.2)	0.248
Hemoglobin (g/dL) ^a^	13.7 (12.4–14.7)	13.1 (1.7)	13.7 (12.7–14.8)	0.8 (0.7–0.9)	0.001
**Lifestyle**					
Physical activity, yes	1902 (67.7)	72 (70.6)	1830 (67.6)	1.2 (0.8–1.8)	0.530
Smoking, former or current	892 (33.8)	36 (35.3)	856 (33.8)	1.1 (0.7–1.6)	0.752
Alcohol intake, occasional or often	1186 (45.0)	49 (48.0)	1137 (44.9)	1.1 (0.8–1.7)	0.531
Coffee, yes	947 (33.7)	33 (32.4)	914 (33.8)	0.9 (0.6–1.4)	0.765
Tea, yes	1742 (62.0)	61 (59.8)	1681 (62.1)	0.9 (0.6–1.4)	0.636
Dietary supplements use, yes	1085 (38.6)	44 (43.1)	1041 (38.5)	1.2 (0.8–1.8)	0.343
Adjusted cholesterol intake (mg)^a^^a^	193.4 (103.6–313.7)	188.1 (110.2–320.3)	194.4 (103.5–313.5)	1.0 (1.0–1.0)	0.360
Adjusted SFA intake (%) ^a^	8.6 (6.0–11.7)	8.4 (6.8–11.6)	8.6 (6.0–11.7)	1.0 (1.0–1.1)	0.297
**Psychological distress, yes**					
BSRS-5 total sore (0–20) ^a^	1.0 (0–3.0)	3.0 (1.0–5.5)	1.0 (0–3.0)	1.2 (1.2–1.3)	<0.001
BSRS-5 ≥ 6	231 (8.8)	25 (25.0)	206 (8.2)	3.8 (2.3–6.0)	<0.001
Sleep disturbance	945 (36.1)	56 (56.0)	889 (35.3)	2.3 (1.6–3.5)	<0.001
Anxiety	551 (21.0)	40 (40.0)	511 (20.3)	2.6 (1.7–4.0)	<0.001
Hostility	703 (26.9)	53 (53.0)	650 (25.8)	3.2 (2.2–4.9)	<0.001
Depression	681 (26.0)	48 (48.0)	633 (25.1)	2.8 (1.8–4.1)	<0.001
Inferiority	452 (17.3)	33 (33.0)	419 (16.7)	2.5 (1.6–3.8)	<0.001

Note

^a^ continuous variables expressed as mean (standard deviation) for normal distribution, and median (interquartile range) for non-normal distribution

SFA, saturated fatty acid; OR, odds ratio; CI, confidence interval; BSRS-5, Five-Item Brief Symptom Rating Scale.

Besides age and sex, univariate analysis suggested 18 other possible variables with *p* < 0.15: ethnicity, marital status, perceived health status, heart disease, hypertension, respiratory tract diseases, gastrointestinal tract diseases, hyperlipidemia, gout, medications for heart disease, hypertension medications, gastrointestinal disease medications, thyroid medications, gout medications, analgesic medications, hyperglycemia, hemoglobin, and psychological distress. The variance inflation factor for each of these variables was <1.5, indicating that the no-collinearity assumption was met. The results of the Hosmer-Lemeshow test on both models, whether BSRS-5 data with continuous or dichotomous variables, showed that they exhibited a good fit (both *p >* 0.05). Furthermore, after adjusting covariates, the BSRS-5 score was associated with AP (OR = 1.2, 95% CI = 1.13–1.26, *p* <0.001) (Table 3, model 1), and the subjects with psychological distress were found to have a higher risk of AP (OR = 2.97, 95% CI = 1.76–4.99, *p* < 0.001) (Table 3, model 2).

**Table 3:** Relationships between various factors affecting AP determined by stepwise logistic regression.

Variable		OR	95% CI	*p*
**Model l**				
	History of heart disease, yes	5.22	3.19–8.56	<0.001
	Gastrointestinal disease medication used, yes	2.49	1.39–4.47	0.002
	Psychological distress, BSRS-5 total score	1.20	1.13–1.26	<0.001
**Model 2**				
	Perceived health status, poor	1.74	1.11–2.75	0.017
	History of heart disease, yes	4.92	2.99–8.09	<0.001
	Gastrointestinal disease medication used, yes	2.29	1.27–4.13	0.006
	Psychological distress, BSRS-5 ≥ 6	2.97	1.76–4.99	<0.001

OR, odds ratio; CI, confidence interval; BSRS-5, Five-Item Brief Symptom Rating Scale.

## Discussion

Our study showed that measures of psychological distress were significantly associated with AP. Furthermore, we also found that a history of heart disease and gastrointestinal medications were independently associated with AP.

These results support the main hypothesis in this study and are consistent with those of previous studies [19,20]. A follow-up study showed that new or persistent psychological distress significantly increased the risk of future AP diagnosis in males [19]. Large-scale research showed a significant correlation between high levels of psychological distress and self-reports of physician diagnosis of heart attack/angina [20]. Psychological distress can significantly increase the risk of myocardial ischemic changes, as determined by exercise ECG [5] or nuclear medical imaging examinations [6]. Psychological distress also increases the frequency of AP occurrence in patients with acute MI [7], which may be related to behavioral factors, biological factors, or their interactions. Negative emotions experienced during periods of psychological distress decrease medication compliance and health-promoting behaviors, thus increasing the risk of metabolic abnormalities and CAD. Psychological distress also affects the biological activity of the hypothalamic-pituitary-adrenal axis, and autonomic nerves, and activates platelets and inflammatory cytokines. This induces endothelial dysfunction and atherosclerosis, causes vasoconstriction, accelerates the heartbeat, and increases the ventricular load, thereby increasing the incidence of AP symptoms [4,34,35]. In addition, interactions between the behavioral and biological factors link psychological distress to angina. For example, poor adherence to physical activity and antidepressant medications may increase biological inflammation and depressive symptoms [35].

Previous studies on different aspects of psychological distress usually considered combinations of various symptoms, such as depression, anxiety, personality traits, functional impairment, and behavioral problems. Depending on the scale utilized, the time frame considered was a 7- to 30-day period prior to the evaluation. Most studies used different cutoff points to distinguish between various levels of individual distress, to screen out high-risk groups, or calculate the prevalence of psychological distress (approximately 5–27% for the general population [1]). BSRS-5 is an easy to use tool, which can be beneficial for rapid screening in the clinical setting. In the present study BSRS-5 was used to assess the occurrence of sleep disturbance, anxiety, hostility, depression, and inferiority over the 7-day period prior to the evaluation. In our sample the prevalence of psychological distress was 8.8%, and approximately half of the subjects with AP experienced sleep disturbance, hostility, and depressive symptoms.

Assessing the overall psychological distress may reduce the probability of overlooking mental health conditions. For example, manifestations of depression include three dimensions—cognitive, somatic, and affective disturbances—which may present different subtypes of symptoms [36]. Hostility is usually accompanied by anger; responses to anger may include withholding or inhibiting anger (anger-in) as well as adopting language or limb aggressive (anger-out) responses [37]. Depression and anxiety exhibited strong correlations with anger-in responses, whereas hostility had a strong correlation with anger-out responses [38], indicating that hostility and depression might represent completely different responses to the same emotion expressed by the same person. Furthermore, insomnia is also related to both hostility and depression [39]. The results of investigations conducted in nine countries showed that the most important chronic conditions affecting sleep were depression and angina, which exhibited a dose-response relationship with the number of chronic conditions [40]. Focusing on only a single symptom, such as depression, might lead to overlooking other significant clinical manifestations, including hostility and sleep disturbances.

We also found that a history of heart disease and the use of gastrointestinal medications were significantly correlated with AP. However, we were unable to confirm the specific type of heart disease or gastrointestinal medication used by the subjects. Patients who use gastrointestinal medications usually have gastrointestinal disease. Heart disease and gastrointestinal diseases may share similar risk factors and symptoms; sometimes both diseases coexist [10]. The presence of AP was assessed using a self-reported questionnaire in this study. As such, we were unable to determine whether the AP was actually caused by cardiac ischemia as opposed to other non-cardiac factors, and it may be difficult for patients to distinguish between different these types of pain. In addition to CAD, other types of heart disease (such as ventricular hypertrophy, valvar disease, and cardiomyopathy) may reduce coronary blood flow, thus causing AP symptoms [41]. The esophagus is stimulated by gastric acid, which induces cardioesophageal reflex, which in turn can cause coronary artery spasm, leading to myocardial ischemia and angina symptoms. Gastrointestinal disorders (such as chronic gastritis, peptic ulcer, etc.) increase platelet aggregation, cause vitamin B12 deficiency, and increase the concentration of homocystein in the blood, promoting the progression of atherosclerosis [10,11]. Gastrointestinal disorders could also be related to alterations in pain perception, potentially leading to increased self-reported angina in patients with psychological distress [42]. This may also explain the link between AP and gastrointestinal symptoms, at least in part. Moreover, subjects with gastrointestinal symptoms but without angina may be unable to distinguish between the two, so this seems a likely explanation for the independent association of gastrointestinal medication with AP. Non-cardiac chest pain may be part of the reason why the results of this study did not show any correlation between some of the ‘traditional’ CAD risk factors (such as diabetes, hyperlipidemia, and unhealthy lifestyle) and AP.

To the best of our knowledge, this is the first study to explore the correlation between psychological distress and AP using population survey data from Taiwan. Population studies with large sample sizes are not limited by patients’ receipt of medical attention or physician diagnoses, thus better reflecting “real-world” situations. However, this study has a number of notable limitations. First, we lacked follow-up data which could help to determine the cause or at least the long-term consequences of the findings, and we were unable to determine causal relationships between variables due to the cross-sectional design of this study. Further, our secondary data analysis was limited by the reliability and validity of the original data; further, NAHSIT data cannot be extrapolated to other regions or ethnic groups. Only data obtained from patients who were both interviewed and subjected to physical examinations were analyzed; thus, we were unable to eliminate sample selection bias. In addition, the questionnaire may have had recall or reporting bias. The duration of AP episodes was not included. This might have affected the reported occurrence of AP. AP was assessed based on self-reported questionnaire responses alone, and the observed relationships between AP symptoms and clinical medical evidence or diagnoses therefore could not be confirmed. We were unable to confirm whether the AP was caused by cardiac or non-cardiac factors. The BSRS-5 questionnaire assessed the occurrence of five parameters of psychological distress over the preceding week, but this information could not identify specific types of psychological distress symptoms. Future studies should use different scales, evaluate different combinations of factors, and extend the evaluation timeframe in order to identify the best practical screening tool.

## Conclusions

Psychological distress was independently associated with AP. We found that the BSRS-5 questionnaire was convenient psychological distress-screening tool for clinical use. These findings suggest that healthcare providers should be aware of the impact of psychological distress on AP, and early mental health screenings may facilitate timely cross-disciplinary treatment and care.

## Supporting information

S1 Table Comparison of basic characteristics between respondents and non-respondents. (DOCX)
