# Supplementary material for: The association between psychological distress and angina pectoris: A population-based study
Source: PLoS One. 2019 Nov 8;14(11):e0224451. doi: 10.1371/journal.pone.0224451 (PMC6839898; doi:10.1371/journal.pone.0224451)
Supplement: S1 Table — (DOCX) [file pone.0224451.s001.docx]

**Supporting information**

S1 Table. Comparison of basic characteristics between respondents and non-respondents

| Variable | |  | Respondents | Non-Respondents | X^2^ | *p* |
| --- | --- | --- | --- | --- | --- | --- |
|  |  |  | *N*=2808 | *N*=1878 |  |  |
|  |  |  | *n* (%) | *n* (%) |  |  |
| Basic characteristics | | |  |  |  |  |
|  | Sex |  |  |  | 1.9 | 0.170 |
|  | | Male | 1375 (49.0) | 958 51.0) |  |  |
|  | | Female | 1433 (51.0) | 920 (49.0) |  |  |
|  | Age (years) | |  |  | 49.9 | <0.001 |
|  | | 19−44.9 | 840 (29.9) | 732 (39.0) |  |  |
|  | | 45−64.9 | 945 (33.7) | 613 (32.6) |  |  |
|  | | ≥ 65 | 1023 (36.4) | 533 (28.4) |  |  |
|  | Ethnicity | |  |  | 0.1 | 0.932 |
|  | | Fukienese | 1612 (57.4) | 1073 (57.1) |  |  |
|  | | Hakka | 572 (20.4) | 379 (20.2) |  |  |
|  | | Other | 624 (22.2) | 426 (22.7) |  |  |
|  | Geographic location | |  |  | 4.4 | 0.113 |
|  | | Northern | 1066 (38.0) | 707 (37.6) |  |  |
|  | | Central/Southern | 729 (25.9) | 445 (23.7) |  |  |
|  | | Eastern/Other | 1013 (36.1) | 726 (38.7) |  |  |
|  | Marital status | |  |  | 48.5 | <0.001 |
|  | | Unmarried | 320 (11.4) | 345 (18.4) |  |  |
|  | | Married with living spouse | 1994 (71.0) | 1190 (63.4) |  |  |
|  | | Other | 494 (17.6) | 343 (18.2) |  |  |
|  | Education | |  |  | 22.0 | <0.001 |
|  | | Primary school and below | 1187 (42.3) | 735 (39.1) |  |  |
|  | | Junior/  senior high school | 1059 (37.7) | 832 (44.3) |  |  |
|  | | College and above | 562 (20.0) | 311(16.6) |  |  |
| Employed, yes (*n*=4676) | | | 1218 (43.9) | 944 (50.4) | 19.2 | <0.001 |
| Economic status, difficult (*n*=4540) | | | 839 (30.9) | 627 (34.3) | 5.7 | 0.059 |
